# A Video Game for Entrepreneurship Learning in Ecuador: Development Study

**DOI:** 10.2196/49263

**Published:** 2023-10-11

**Authors:** Esteban Crespo-Martinez, Salvador Bueno, M Dolores Gallego

**Affiliations:** 1 Department of Business Organization and Marketing Universidad Pablo de Olavide Seville Spain; 2 Centro de Investigación, Innovación y Desarrollo de Tecnologías Aplicadas y Emergentes - CIIDTAE Universidad del Azuay Cuenca Ecuador

**Keywords:** video game, computer game, serious game, Unity 3D platform, entrepreneurship, business simulator

## Abstract

**Background:**

Games have been a part of human life since ancient times and are taught to children and adults who want to simultaneously have fun and learn. Nevertheless, in the third decade of this century, technology invites us to consider using video games to learn topics such as entrepreneurship. However, developing a serious game (SG) is difficult because everyone who forms part of the game development team requires adequate learning resources to acquire the necessary information and improve their game development skills.

**Objective:**

This work aimed to detail the experience gained in developing ATIC (Aprende, Trabaja, Innova, Conquista [learn, work, innovate, conquer]), an SG proposed for teaching and learning entrepreneurship.

**Methods:**

To develop a videogame, first, we established a game development team formed by professors, professionals, and students who have different roles in this project. Scrum was adopted as a project management method. To create concept art for the video game, designers collected ideas from various games, known as “getting references.” In contrast, narratology considers the life of a recent university graduate immersed in real life, considering locations, characteristics, and representative characters from an essential city of Ecuador

**Results:**

In a Unity 3D video game in ATIC, the life of a university student who graduates and ventures into a world full of opportunities, barriers, and risks, where the player needs to make decisions, is simulated. The art of this video game, including sounds and music, is based on the landscape and characteristics of and characters from Cuenca, Ecuador. The game aims to teach entrepreneurs the mechanisms and processes to form their businesses. Thus, we developed the following elements of an SG: (1) world, (2) objects, (3) agents, and (4) events.

**Conclusions:**

The narrative, mechanics, and art of video games are relevant. However, project management tools such as leaderboards and appointments are crucial to influencing individuals’ decision to continue to play, or not play, an SG. Developing a serious video game is not an easy task. It was essential to consider many factors, such as the video game audience, needs of learning, context, similarities with the real world, narrative, game mechanics, game art, and game sounds. However, overall, the primary purpose of a serious video game is to transmit knowledge in a fun way and to give adequate and timely feedback to the gamer. Finally, nothing is possible if the members of game development team are not satisfied with the project and not clear about their roles.

## Introduction

### Background

Entrepreneurship has become a crucial economic activity because it is a pathway to escape poverty and revitalize stagnant economies [1,2]. However, the COVID-19 pandemic has revealed informal workers’ precarious and vulnerable conditions [3,4]. The sustainable development goals (SDGs) adopted by the United Nations in 2015 aim to enhance economic growth, social inclusion, and environmental protection worldwide [5]. SDG 4 focuses on designing strategies to lift people from poverty, including developing competencies for the SDGs in business administration education [6]. The pandemic has deepened the education crisis [7], leading teachers and students to explore new teaching and learning methods using technology. Electronic learning platforms, massive open online courses, comprehensive communication and collaboration tools such as Zoom, and video games are transforming university education and optimizing students’ learning quality [8-15]. However, electronic learning can become tedious, leading to distractions for most students.

As described by Antonaci et al [16] and Bellotti et al [17], serious games (SGs) have proven to be a learning method for conveying skills on complex tasks by incorporating sound learning and pedagogical principles into their design and structure. However, SG development is complex [18]. There are a few things to consider, such as the narrative [19], the artistic elements, or the playability [20,21], all running together inside a video game engine [22]. As in the case of software development, this critical activity involves various aspects, such as development standards. Standards such as International Organization for Standardization (ISO)/International Electrotechnical Commission (IEC) 12207 or ISO/IEC 29110 help companies to manufacture software products with an adequate quality level. These standards are relevant in software engineering because they engineering principles are involved in the software’s design, development, and maintenance [23].

Education to teach the processes inherent to innovation and entrepreneurship is vital in professional training, which have an impact on macroeconomic variables such as economic growth and the development of an economy [24]. SDG 17 (“Partnerships for the goals”) is receiving the most attention from nations in terms of harnessing digital technology to accelerate progress toward achieving the SDGs by 2030. It refers to an alliance and collaborative partnerships [25-27]. As a contribution to the scientific society promoted by the University of Azuay, Ecuador, and the University of Pablo de Olavide, Spain, a research project on learning entrepreneurship through SGs (ie, a video game on the Unity 3D platform) was proposed. The project is in the development stage.

This paper aims to detail the experience of constructing ATIC (Aprende, Trabaja, Innova, Conquista [learn, work, innovate, conquer]), an SG for entrepreneurship learning that considers the Latin American environment. ATIC is the phonetic equivalent of “atiq,” which means “the one who can do everything” in Quechua—the language spoken by the Inca people and the native language that has spread the most throughout Latin America. ATIC allows the player to gain experience evaluating future scenarios and their implications, a topic related to anticipatory competence. This study aims to solve the research question of what characteristics an SG should have for people to learn entrepreneurship.

The most relevant portions of this work include presenting the results of applying a methodology that describes the experience of forming a work team of professors and students; the roles and responsibilities of each team member; and the elements considered in constructing this SG, including the narrative and game mechanics. A simulated virtual environment such as that of a video game provides controlled simulation, making it advantageous for learning entrepreneurship.

This paper is structured as follows. The next subsection provides state-of-the-art related works, with the application and use of procedures for developing video games in the educational context. The *Methods* section presents the methodology used to obtain the results, which are presented in the *Results* section. Subsequently, we present the *Discussion* section, in which, among other things, we address the implications of the findings and possible directions for future research.

### State of the Art

The video game industry is an ever-growing sector, with a projected global market size of US $293.6 billion by 2027 [28,29]. Game developers are increasingly turning to software development methodologies to improve the efficiency and quality of their development processes to keep up with this growth. Such methodologies enable game developers to manage complex projects efficiently, with clear goals, schedules, and resources. Agile and scrum methodologies provide a framework for continual improvement, allowing developers to iterate on game mechanics, graphics, and other features throughout the development process, ultimately reducing costs and increasing quality.

Software development is a specialized field [30] encompassing various tasks, such as identifying user requirements, planning and distributing tasks, and selecting resources and languages [31,32]. However, these tasks must be set into a standard framework to assess quality [33,34]. In this way, as explained by Musil et al [35], similar to traditional software engineering projects, video game development includes processes for project planning and control, constructive methods for artifact creation, and analytical methods for verification and validation purposes. In this respect, some software engineering standards have been identified.

ISO/IEC 12207 [36] and ISO/EIC 29110 [37] are international software engineering standards that provide a common framework for software life cycle processes for very small entities. A very small entity is an organization, enterprise, department, or project in which a maximum of 25 persons work together [38]. ISO/EIC 15504 [23] (now ISO/EIC 33060:2020 [39]) provides a framework for system life cycle processes. In project management, creating defined processes, product quality, efficacy, and efficiency are desirable characteristics of a software development team [40,41].

Developing specific SGs, such as educational games, presents unique challenges [42]. Short SG development processes must have pedagogical, technical, and integration aspects [43]. Video games and SGs require a game engine to work, and Unity 3D and Unreal Engine are the preferred game engines for training and educational applications [44-47].

The viewpoint expressed by Pinar [7] highlights the challenges faced by the education sector owing to the COVID-19 pandemic, leading to the adoption of technology-based learning solutions. Bellotti et al [17] and Juzeleniene et al [48] discuss using SGs to promote entrepreneurship among higher education students and managers of small and medium enterprises. Almeida [49] presents a cloud-based SG that offers learning content related to entrepreneurship, whereas Jauregui Sánchez et al [50] propose *Acción Capital*, an SG aimed at teaching key concepts about entrepreneurship using the design thinking methodology. Finally, Ahmad et al [51] present a framework for developing and launching a successful video game, including the concept stage, development budget, marketing strategies, and downloadable content.

## Methods

### Preparing Students to Join the Game Development Team

The game development team is essential [52,53]. As suggested by Kletenik and Sturm [32], the first step is to motivate students to become part of this game development team and include video game development courses in computer curricula [54]. The content should include, at a minimum, game genres, sprites, movement, assets, animation, collisions, and user interaction [55], but narrative, games theory, and playability design are essential topics to consider [56,57]. Hence, the optative course of Computer Science Engineering was proposed by the Center for Research, Innovation, and Development in Applied and Emerging Technologies (*Centro de Investigación, Innovación y Desarrollo de Tecnologías Aplicadas y Emergentes* [CIIDTAE]) at the University of Azuay in Cuenca, Azuay, Ecuador. The program includes the following macrotopics: (1) the history of video games; (2) gamification; (3) preproduction techniques; (4) game production; (5) storytelling, which includes narrative creation; (6) video game design (first level); and (7) video game art.

In addition, the following individual courses, as part of the training process, were proposed: (1) narrative creation, (2) digital 3D object development, and (3) Unity 3D video game engine. The first 2 were proposed and to be taught by professors, and the last was proposed by a student.

### Forming the Game Development Team

To develop a video game, it is necessary to establish some roles in the preproduction phase. In this case, the following roles were set up: (1) the game designer, who assumes the responsibility of defining the user experience in the game; (2) the game developer: in this case, the students, whose responsibility is to write the video game code; (3) the game producer, who is responsible for the video game marketing; (4) the game administrator, who is responsible for the data administration and project continuity; (5) the game artist: in this case, 1 professor, 1 designer, and the students who have participated in the digital 3D design course; and (6) the game writer, who is responsible for creating the narrative. A total of 57 persons are part of the game development team. Table 1 presents the game development team distribution.

The scrum agile method and Milanote were used for project management, with 1-week sprints and effective communication through Milanote’s easy-to-use visual board tool. Milanote also enhanced quality assurance [58,59].

**Table 1:** The number of persons who were part of the serious game development team (N=57).

	Professor or researcher (n=9), n (%)	Professional (n=4), n (%)	Student (n=44), n (%)
Game designer^a^	5 (56)	N/A^b^	2 (5)
Game developer	1 (11)	3 (75)	7 (16)
Game producer and game administrator^c^	1 (11)	N/A	N/A
Game artist	1 (11)	1 (25)	30 (68)
Game writer	1 (11)	N/A	5 (11)

^a^The 5 researchers from the project assumed the role of game designer.

^b^N/A: not applicable.

^c^The same person assumes the role of both game producer and game administrator.

### Video Game Graphic Art

In developing a video game, the visual aspect is critical for creating an immersive and visually appealing experience. Ambrose et al [60] discuss the various stages involved in establishing a graphic line, which includes collecting and classifying graphic counterparts, concept design, and graphic line incorporation. The first step involved grouping the graphics into an interface, gameplay, general aesthetics, chromatic colors, graphic style, and morphologies of characters and scenarios. This classification helped in building a permanent consultation resource for the design team.

To create concept art for the video game, designers collected ideas from various games, known as “getting references.” Three reference styles were obtained from Firewatch [61], FIFA 2022 [62], and The Legend of Zelda: Breath of the Wild [63]. The concept design stage involved determining the game’s general aesthetics and designing 3 graphic proposals based on the analyzed aesthetics and graphic languages. The proposals were validated by the professionals and experts on the team, leading to a consensus that made it possible to build the different graphic languages to be incorporated.

The aggregated graphic line responds to simplified morphologies with rounded edges dominated by vertical lines. It reflects a language related to constant movement and actions. The selected aesthetic responded to dynamic geometries and proportions, adapting to the scale of the characters and scenarios. In addition, some elements were rescaled to build visual hierarchies and guide the observer’s gaze toward more profound, eloquent, and poignant messages. This approach helps create a visually immersive and appealing experience for the player.

### Sound Effects

Musicians have 2 primary roles in video game development: to create sound and musical effects for video games and to apply sound and music creation tools to video game development [64]. Early technology limited composers to 8-bit themes [63], but technology has become more complex [65], and composers have expanded the boundaries of video game music as a genre.

According to Rone [66], the music accompanying the opening peritexts in 2 Legend of Zelda games reflects their reception history and continuity within the series mythology. Thus, the music accompanying the peritext enables players to engage in Zelda’s potential for self-reference. Under this concept, music becomes essential in the game because it provides sensations to suit the environment [67].

The background sounds proposed for ATIC include the noises of the city of Cuenca, a space where entrepreneurs will develop their business activities. An omnidirectional microphone was used to capture the contextual sounds, which were then corrected using Adobe After Effects.

### Narrative

In narratology, the narrative comprises the story, a series of related events, and the discourse [63,68], similar to the version used in cinematic or literary creation. However, in a video game narrative, interactivity is the distinguishing feature [68-70]. A video game’s narrative must highlight specific skills, such as immersion, identification, and reward, to attract players and ensure their engagement [71,72]. A video game’s narrative provides entertainment and fun, which are its fundamental purposes [71,73,74].

The narrative designer must consider the game’s initial aspects in an educational context. Students are provided with clarity on the main focus of the SGs, giving them a clear starting point and means to structure their game [32,73]. The game must contain 4 elements: the world, objects, agents, and events [75]. The game world can be linear, multicursal, or open, and objects can be dynamic, user created, or static [75,76]. The sequence of events can be selectable, open, or plotted, and the narratological notion of nuclei and satellites is vital [75].

The narrative writer presents the first-level narrative of an entrepreneurship video game. At the beginning of ATIC, the player must answer 20 questions to set the avatar’s profile, enemies, and challenges. The identified entrepreneurial characteristics in the avatar’s personality test are *proactive*, *persistent*, *risk taker*, *planner*, *persuasive*, and *safe* [77,78]. The game narrative was designed using the Twine open-source tool to tell an interactive-nonlinear story and a linear story with ramifications [79]. The narrative immerses the player through microgames that provide natural and necessary information for starting a business in Cuenca city. The use of locations and characters representative of the city allows the player to identify with the environment and the processes they should consider undertaking in real life. The game’s reward is reflected in the consumers’ response, the profits obtained, and the challenges overcome that will allow the player to advance in level and meet the proposed objectives.

### Game Mechanics

The work of Brooks and Brooks [80] discusses the mechanics and motivational factors of built-in games, which are both intrinsic and extrinsic. According to Larsen and Schoenau-Fog [68], game mechanics are the core of the interactive part of a game and inherently tell a narrative through its mechanics. DuBravac [81] identified 7 basic game mechanics: achievement, appointment, rewards, leaderboards, privacy, social engagement loops, and modifiers. Knysh et al [82] add immediate feedback, points and leaderboards, narratives, missions, quests, competition, and compensation as essential elements of the last decade’s SGs.

According to DuBravac [81], the achievement mechanic is vital in teaching entrepreneurship skills, and players must move up levels, gain badges, and earn points to show progression. The appointment mechanic rewards players for returning at specific times, generating use continuity intention. Rewards are essential to incentivize players, and questing clarifies the task to be completed. Leaderboards should be simple and rank only the top few for each ranking category. Privacy is crucial, and all information displayed on the leaderboard will be accessed only by the player and the teacher, following Ecuador’s Organic Law of Personal Data Protection [83]. Viral mechanics engage players in play, and when they share awards or invite other friends to play the game, they receive additional awards. Modifiers, which change gameplay or functionality, are add-ons to a game that students can use to increase their achievements or rewards [84].

To collect ideas for modifiers, the ATIC working team played various games, including (1) The Lemonade Business [85]; (2) Grand Theft Auto V [86]; (3) FIFA 2022 [87]; (4) Monopoly; (5) Startup Empire–Idle Tycoon [88]; (6) Cook, Serve, Delicious! 2 [89]; and (7) Sim Companies [90]. These games allowed the team to identify new modifiers to incorporate into ATIC, such as winning a lottery or receiving an inheritance to earn money faster.

### Game Engine

Initially, to develop ATIC, the Construct 3 platform was considered. This system provides a *drag and drop* interface to facilitate game development using a set of building blocks (similar to the building blocks that children use to construct objects [91]), assisting users who have no programming skills [42,92]. ATIC was first designed to be an arcade game in 8 bits styled as a 2D game [93]. However, as the game began to develop, the game programmers considered the Construct 3 platform insufficient to manage multiple parallel events.

Thus, Unity 3D was considered the best engine for this project. One of the reasons for selecting it, as described by Haas [45], is that Unity 3D can deploy to a wide variety of target platforms using the same code and assets. Another reason is that Unity 3D and Unreal Engine are two of the most used game development engines, aggregating millions of registered users and being used to develop many commercial games, and, of course, the community support level is higher [94].

Technological media is another component to consider. According to Alonso-Díaz et al [95], PCs are the most used hardware for running video games. Under this precept, and considering this SG’s primary target, a PC is the better platform to run ATIC.

### Ethical Considerations

Video games can be a powerful tool when used in the correct context, and they have proven to be an effective way to engage users in areas that traditional methods cannot, making things more fun, unique, competitive, and interesting [96]. Although many of these applications are designed to benefit the user, some have potential ethical pitfalls or just manipulate players outright [97].

According to Ecuador’s Organic Law of Personal Data Protection [83], personal user data confidentiality must be guaranteed through anonymization. Although Ecuador has an Organic Law on Personal Data Protection, it does not explicitly consider information collection scenarios involving video games or information collection through video games, and the informed consent of the person involved regarding their personal information is not regulated [98]. Relatedly, in the study conducted by McKelvey [99], most students stated that they would consider legal and ethical issues and society’s views when developing computer games.

In the design of a video game, some aspects, such as persuasion and push techniques, influence and change attitudes and behaviors. Here, ethics becomes relevant because it should be considered in the persuasive aspect of the design. Some levels of transparency are required. The users must be informed about the persuasive intent of the technology [100]. According to Sandovar et al [101], an ethical perspective must inform (1) the cultural artifact, clarifying whether the game is good or bad; (2) the business ethics, referring to creating the game ethically; (3) the ethical play, which means fair or ethically relevant experiences for players; and (4) the ethical framework, referring to defined actions for the player. In this way, a consent agreement on data collection and transparency in operation must be considered [83,102].

### Resource and Budget Planning

This is a project of the CIIDTAE laboratory at the University of Azuay. All financial resources were provided by the research department of the University of Azuay. The budget for developing this video game provides for (1) developers’ salaries, (2) designers’ salaries, (3) researchers’ salaries, (4) development licenses, and (5) publication fees for scientific events. Publication fees were sponsored by the University of Pablo de Olavide.

Some requirements had to be met to formalize the research project: (1) compliance with ≥1 objectives of Ecuador’s National Development Plan 2019-2021 [103], (2) compliance with at least 1 of the SDGs [25,27], and (3) a description of the type of impact expected to be generated by the project (social, economic, scientific, among others).

## Results

This section presents the results of the experience of designing and constructing ATIC, considering the video game graphic art, sound and effects, narrative, game mechanics, and resource and budget planning.

### Game Development Team Configuration

As described in the *Methods* section, 57 persons are part of the game development team. According to Aleem et al [104], (1) game design document management, (2) game engine development, (3) game test management, (4) programming practices, and (5) team configuration management become the most significant tasks to take into account. Keeping this in mind, the ATIC project game development team consists of the following roles: (1) game designer, (2) game developers, (3) game producer, (3) game administrator, (4) game artist, and (5) game writer. The project documentation is maintained in a shared Milanote worksheet and a shared Google Drive folder.

### Video Game Graphic Art

**Figure 1:**
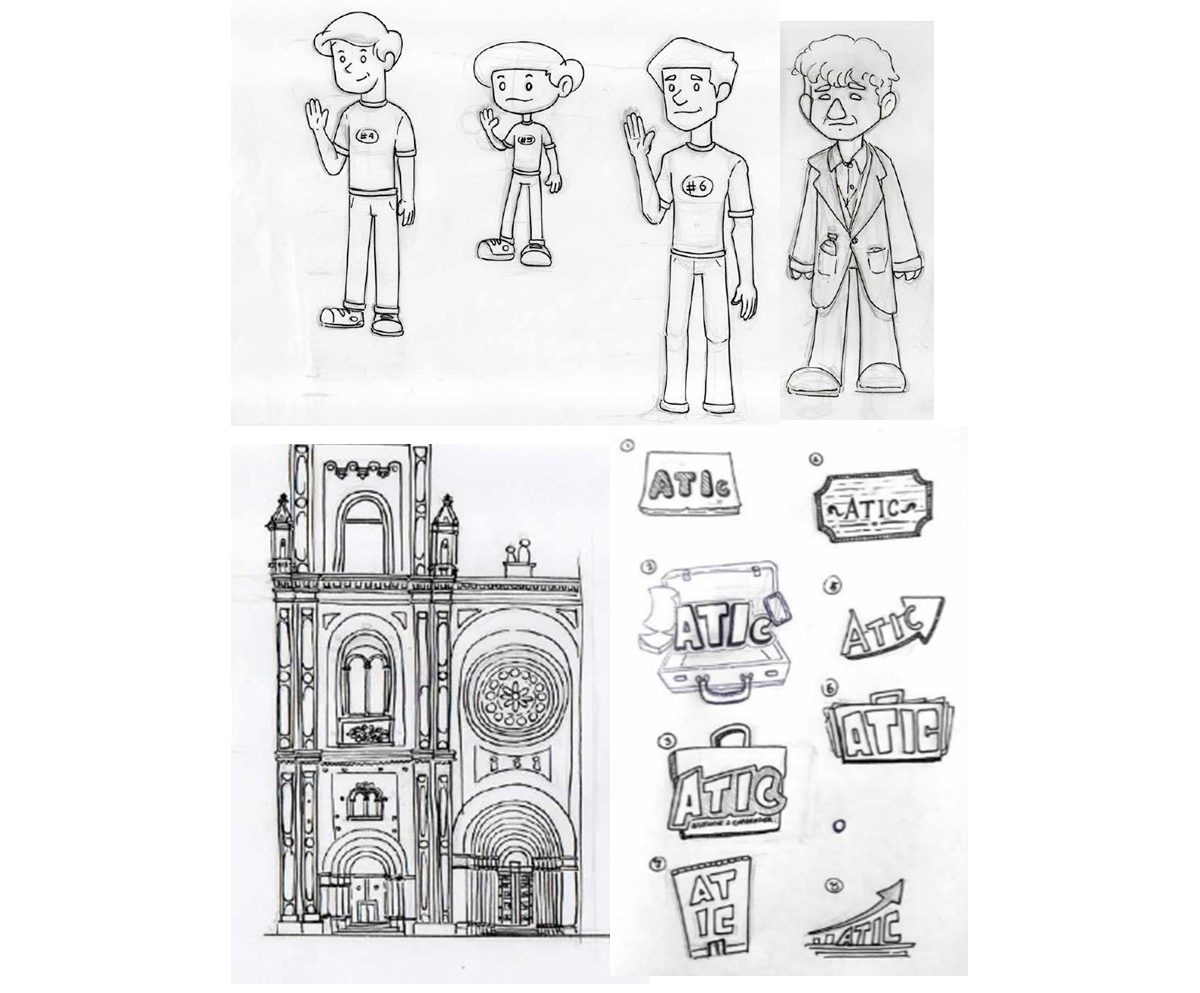
Video game art sketch.

In the concept design stage, the designers made sketches using paper and pencil, inspired by some characteristics of Cuenca city, such as the city’s colonial buildings or well-known personages. Figure 1 shows (1) different versions of the avatar, (2) a representative building from Cuenca city, and (3) the video game trademark.

As for the selected colors, unsaturated tones are preferred, allowing color palettes with harmonious relationships to be used. In some cases, incorporating complementary colors facilitates the contrast among the navigation interfaces concerning the backgrounds.

Different graphic decisions allowed us to establish a contemporary graphic line according to the aesthetics of some video games related to the current scenario. The geometry of the environments abstracts and stylizes the main features of Cuenca, allowing its identification through a quick and simplified reading of some of the most emblematic buildings and incorporating some of the city’s iconic characters.

**Figure 2:**
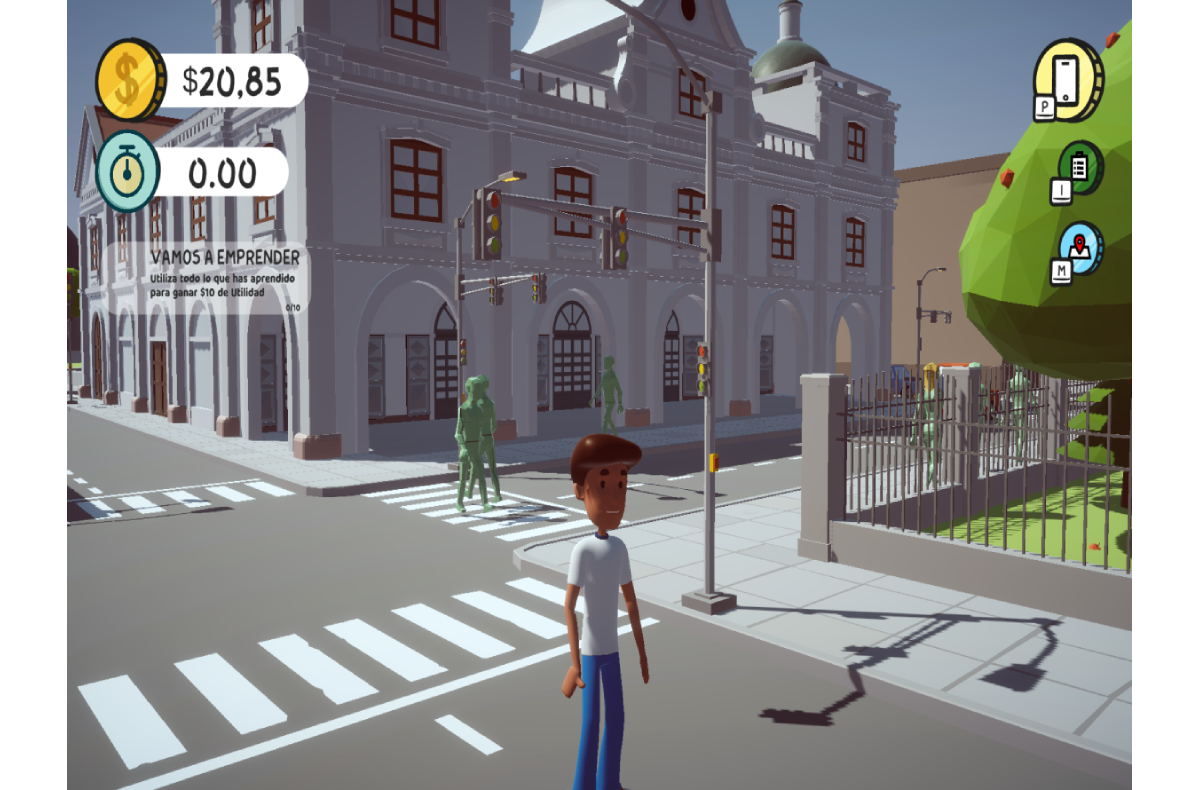
Video game final art object.

Figure 2 shows one of Cuenca’s most representative buildings, the Old Cathedral or Church of the Tabernacle, built in the 16th century. The figure also shows Joshua, the character representing the entrepreneur. Joshua has a challenge to meet: start a business with the capital that he has managed to obtain (US $20.85) in a specific time period, both of which are presented in the upper left portion of the screen. In the upper right portion are icons representing quick access to the inventory, the financial and reputational statistics, and the city map, where the player can find the destinations for carrying out the proposed actions.

Here, the visual system allows for establishing relevance and identification, thanks to the features that determine the typology of the buildings and the characters. Next, we proceeded to model the wireframes that will allow navigation in the video game, providing easy-access scenarios and visual cleanliness in the selection and access to different game sections. The video game logo manifests itself as an identifier that simplifies, through its geometry, the most significant elements included in an enterprise that are daily recognized and associated with objects belonging to the workplace and businesses.

In ATIC, players can select avatars representing different ethnic groups in the region, enhancing their identification and representation. The character sheet identifies each avatar’s physical, psychological, and behavioral characteristics. Unlike the work done by Jauregui Sánchez et al [50], in which no avatars are available, the authors highlight the relevance of avatar creation and customization, including options for uploading a custom avatar, configuring different aspects of the avatar, generating an avatar from a photograph, and considering assets supporting the user’s identification with the avatar. Other relevant elements include age control, body parts, avatar size, and tracking to represent movement and gestures. The use of Blender to develop 3D art objects is also highlighted [105]. Emphasis is laid on the importance of avatar design to enable real-time communication with the platform and with other players and to enhance players’ identification and representation [106].

### Video Game Sound Effects

The sound effects for ATIC are based on background noises from the historic center of Cuenca, including (1) the murmur of people buying roses on the square near the Old Cathedral, (2) the sound of the tram as it makes its way through the city, (3) birds singing in the central park in front of the Old Cathedral, (4) the noises along the ravine of Tomebamba river, and (5) some music from street musicians. Other sound effects, such as those associated with coins, drinks, payments, scratches, and car beeps, were sourced from the Open Game Art website [107].

Regarding music, the player can select different styles of music, an option similar to that of the Grand Theft Auto game. In the case of ATIC, music was performed by 2 researchers and 2 students, who demonstrated their ability in this artistic field.

### Narrative

An SG is designed for a primary purpose beyond pure entertainment, and this influence on players’ cognitive, emotional, and social domains increases learners’ motivation and engagement [108]. In their study, Guillén-Nieto and Aleson-Carbonell [109] highlight the game features to support learning effectiveness, the process through which games engage learners, and the types of learning outcomes that can be achieved through gameplay. The narrative, a part of a video game, flows under the author’s direction, whereas interactivity depends on the player for motive power [71].

Considering the relevance of the narrative, as described by Khaled and Vasalou [110], the participatory design introduces new opportunities for SG designers and players. Here, the ATIC game development team allowed the generation of multiple essential ideas for the narrative based on their academic and personal experiences in entrepreneurship.

One crucial lesson about designing for participatory transformative experiences that emerged from the work by Tanenbaum [111] is the importance of a well-specified player character. In ATIC, the character represented by an avatar reflects a person’s personality through their identification with an ethnic and sex group in a context that resembles the reality of a well-known city in Ecuador. For the story to come to life, the narrative must be adequately designed and developed, considering the target audience of this video game.

The gameplay in ATIC is based on a linear narrative with ramifications. The player has several options to choose from, and their choice influences the game’s story, causing the messages and challenges to vary depending on the decision made. However, although the endings could be different, the main story is the same for all players. In addition to providing greater dynamism to the gameplay, the insertion of cinematics and microgames within the main story offers real learning of the conditioning factors of each entrepreneurial scenario in the local environment, generating informative content. It becomes an added value to this simple entertainment.

The scenic environment consists of three scenarios: (1) the entrepreneur has to go looking for seed capital and perhaps work for it or do something to obtain it; (2) the entrepreneur hails from a wealthy family and probably does not need to go to a bank to withdraw funds or work to obtain the seed capital; and (3) the entrepreneur starts with a small sum as seed capital and needs a bank loan to obtain the rest of the capital.

**Figure 3:**
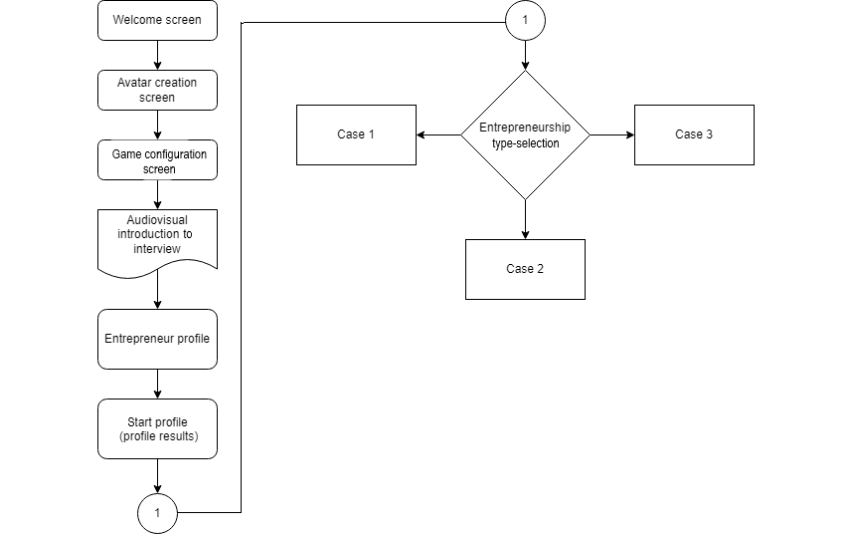
Narrative flow diagram.

In this first release of the game, there are three business scenarios: (1) sell fast food as an itinerant seller, walking the streets in search of customers to discover their preferences and the areas of most significant impact so that at the end of the level players have gained experience in terms of the future of the business; (2) start a street food cart, commonly found at massive affluence events such as music festivals, allowing the entrepreneur to gain more experience and prestige; and (3) start a restaurant, in which food industry knowledge is essential. The narrative is presented in Figure 3 in a flow diagram designed in Twine. Unlike this work, the study by Carlon et al [79] used this tool to design a nonlinear narrative for a video game.

The narrative in ATIC considers the following: whereas the narrative framework focuses on the reasons given for the player’s actions within the game’s story, the conditions of the upgrade mechanisms make how well the player plays more critical within the game; by contrast, if the narrative framework is more effective than the upgrade mechanisms, players will be directed toward helping others or taking more responsible action to achieve a goal or task [112].

### Game Mechanics

The development of video games and software applications share similar traits, such as improving user experience, and an affective design is a critical aspect of video game design to evoke emotional responses and interactions [113]. The game mechanics of a video game include achievement, appointment, rewards, leaderboards, privacy, social engagement loops, modifiers, immediate feedback, points and leaderboards, narratives, missions, quests, competition, and compensation. In a 3D virtual space game such as ATIC, players can move their avatars in multiple directions to find business opportunities and customize their keyboard combinations according to their preferences. The appointment mechanic consists of attending to customer requirements, which accumulate while the player is not playing the game. Players are rewarded with coins and reputation levels, positive or negative, depending on their decisions. The Monopoly game served as inspiration for some of these mechanics, such as the use of random rewards. By contrast, the study by Jauregui Sánchez et al [50] focused on a financial learning game with a linear narrative where the player answers a series of questions related to market capitalization scenarios to promote their businesses.

In ATIC, the player wins the game when he has managed to exceed a set goal, which is made up of a set amount of money, a positive reputation, and a market share achieved. The initial values are established by the system based on the type of entrepreneurial personality identified at the time of creating the avatar. However, these values can be adjusted by the player or the entrepreneurship class teacher who wants to assess the knowledge acquired in the subject.

### Game Engine

The video game constructed by Jauregui Sánchez et al [50] is developed in HTML5 because it integrates the interactive narration developed in Twine. The platform used is the web, accessible via a laptop computer with a Windows operating system and a browser. By contrast, Lin and Pryor [10] used Unity 3D to construct the game Digital Exhibition Space, which allows students to learn collaboratively within their study groups and subsequently with the whole class.

As described previously, ATIC was developed in Unity 3D. The reasons for using this platform are as follows: (1) it provides a better user experience, (2) it allows scaling to ubiquitous technologies such as virtual reality, (3) it allows the exporting of the video game to multiple platforms, (4) it allows the collection of crash and exception reports as well as user feedback so that players can better diagnose issues, and (5) it has the support of a prominent developer community [114].

### Player Engagement

ATIC incorporates the following elements to engage the player and make the learning process more enjoyable and effective: (1) a well-designed storyline relatable to real-life scenarios with clear objectives and challenges to overcome [115]; (2) business simulation exercises such as meeting all legal requirements to establish a business or control the inventory in a production line; (3) interactivity with nonplayable characters (NPCs) [116], who simulate customers’ behaviors (here, decisions are challenging, and the consequences are visible to the player); (4) immediate feedback on their progress [117]; (5) real-life examples [48], considering different business models and situations.

### Resource and Budget Planning

This project complies with the following objectives of Ecuador’s National Development Plan 2019-2021: (1) to guarantee a dignified life with equal opportunities for all people and (2) to promote productivity and competitiveness for economically sustainable growth in a redistributive and supportive manner. In addition, this project aligns with the following SDGs [25]: (1) quality education, (2) decent work and economic growth, and (3) partnerships for the goals.

The effort in this investigation, which was conducted through the partnership between the University of Pablo de Olavide and the University of Azuay, was relevant to obtain the final product. Furthermore, regarding this point, several options for linking with the community will be presented through chambers of commerce, nonprofit foundations, municipal departments, and entrepreneurship promoters.

## Discussion

### Contribution of This Work to Educators and Entrepreneurship Curricula

ATIC could contribute to educators and the entrepreneurship academic curricula by (1) complementing the academic curricula [118] because ATIC teaches entrepreneurship skills in an interactive and attractive manner, and professors could play the game and reinforce concepts and skills taught in the classroom; (2) increasing the interest and motivation of the students through a fun and attractive way to learn, leading to greater participation and engagement in class [119]; (3) providing students with hands-on experience in making business decisions through practical experience [120]; (4) teaching essential skills for entrepreneurship, such as decision-making [50], problem-solving [121], resource management [122], and innovation [123]; and (5) encouraging collaboration and teamwork [124] because students can work together in the game to develop strategies and make business decisions.

### ATIC Versus Other Business Simulation Games

Here, we discuss various business simulation games and their effectiveness in teaching business concepts and strategies. A recent review mentions that an ideal business game must consider combining the following analytical tools: (1) Heptalysis; (2) CATWOE; (3) VPEC-T; (4) SCRS; (5) MoSCoW; (6) MOST; (7) failure mode and effects analysis; (8) fault tree analysis; and (9) Ishikawa’s fishbone diagram, which present different perspectives across business areas [125]. Transform@ is a game oriented toward increasing players’ competence in electronic commerce business in a rural context [126]. Other business simulation games, such as BattMan, Venture Creation Game, CareMe, and Leadership Capacity, are designed to teach different aspects of business models [127,128]. Acción Capital is a game focused on decision-making in a newly created business during a financial crisis [50]. Simventure is an entrepreneurship simulator [129], whereas IT Corp Tycoon focuses on an IT corporation [130].

ATIC considers not only the urban context but also ethnicities, native language, and gender aspects. ATIC is a platform game and will consider the rural context in the future but currently focuses on social entrepreneurship. On the basis of the notion that platform video games and role-playing video games are a genre of video games that is characterized by having to perform an action on a series of platforms and cliffs, facing challenges while collecting objects to complete the game [19] and immersing in some well-defined world [105], it can be said that ATIC belongs to this genre. In summary, ATIC is a unique game because it involves different types of businesses and incorporates microgames within the platform, unlike other business simulation games.

Simulation games have shown promising results in teaching business concepts and strategies. Further research could investigate the effectiveness of ATIC and other simulation games in teaching business skills and fostering entrepreneurship.

### Limitations and Future Works

At present, only the alpha version of ATIC is available, and it does not allow multiple players. This feature will be considered in the next project stage. In addition, ATIC will be brought into virtual reality for interaction in metaverse environments in future stages. The work described in this paper is related to the application of this video game in the classrooms of the University of Azuay to identify the entrepreneurial student’s behavior concerning this gamified scenario. The user interaction results will be described in the next project stage.

### Conclusions

The ATIC video game is the result of research conducted in response to the question of how best to teach entrepreneurship skills in a confined environment, caused by the COVID-19 pandemic in this case. Keeping in mind that the objective of this work is to describe the experience gained in the development of ATIC, the following paragraphs reflect our conclusions.

Regarding recent work trends and economic growth, de la Torre et al [26] suggest that, in this decade, the competence dimensions for achieving the related vital competencies for sustainability are knowledge, skills, and attitudes. This work, related to the development of the ATIC video game, proposes that knowing about other cultures is a factor in obtaining knowledge competence. At the same time, SDGs 4 and 8 are concerned with setting goals and priorities by selecting and distributing tasks and resources, encompassing time management, organization, responsibility, and self-reliance [26,27]. In this regard, the ATIC project contributes, through an SG, to developing strategically integrated problem-solving as a key competence for sustainability.

Developing a video game is not an easy task. As seen in this work, it is essential to be clear about the game’s objective from the beginning. Next, the selection of the game development team from among those eligible will depend on the skills they have to be able to play the different roles allotted to them in the project. In addition, it is relevant to consider that developing a business simulation video game, although it is software, involves elements that developing business software does not involve, such as narrative creation.

The narrative is crucial in a video game, underlying the interaction. Here is where game mechanics becomes essential. The game mechanics design must include the following elements: achievement, appointment, rewards, leaderboards, privacy, social engagement loops (or viral mechanics), and modifiers. Other elements, such as immediate feedback, points and leaderboards, narratives, missions, quests, competition, and compensation, become part of the mechanics.

With regard to the conception of the immersion through microgames provides natural and necessary information for starting a business in Cuenca. In the same way, the use of locations and characters representative of this city allows the player to identify with the environment and enables them to contemplate undertaking a business venture in real life. Furthermore, the reward is reflected in the consumers’ response, the profits obtained, and the challenges overcome that will allow the player to advance in level and meet the proposed objectives.

For game continuity to be maintained, events such as waiting for customer requirements that have not been met will appear as part of the appointment mechanism on the platform, enabling daily contact with the game. Another way to maintain continuity is by generating income in the *bank account* of the entrepreneur in the game.

This project aligns with SDGs 4, 8, and 17. First, with regard to SDG 4, through this game, people can try their hand at entrepreneurship several times until they achieve the objective, helping them to rise out of poverty. Second, in alignment with SDG 8, ATIC helps to develop strategic problem-solving as a key competence for sustainability. Third, the partnership between the University of Pablo de Olavide University, Spain, and the University of Azuay, Ecuador, aligns with SDG 17.

Finally, the development goal of the game has been achieved. A committed team has produced the narrative, the game mechanics, and the other components, as explained in this paper. Although there are some details that need to be refined, it is expected that the beta version of ATIC will be available in October 2023 to continue the investigation around a general question: is it possible to learn to undertake a business venture in Ecuador through SGs?

## Abbreviations

ATIC: Aprende, Trabaja, Innova, Conquista (learn, work, innovate, conquer)
CIIDTAE: Centro de Investigación, Innovación y Desarrollo de Tecnologías Aplicadas y Emergentes (Center for Research, Innovation, and Development in Applied and Emerging Technologies)
IEC: International Electrotechnical Commission
ISO: International Organization for Standardization
NPC: nonplayable character
SG: serious game
SDG: sustainable development goal
